# Effect of COVID-19 Pandemic on Presentation of Patients With Diabetic Retinopathy in a Multitier Ophthalmology Network in India

**DOI:** 10.7759/cureus.19148

**Published:** 2021-10-30

**Authors:** Anthony V Das, Raja Narayanan, Padmaja K Rani

**Affiliations:** 1 Department of eyeSmart Electronic Medical Record (EMR) and AEye, L. V. Prasad Eye Institute, Hyderabad, IND; 2 Department of Vitreoretinal Diseases, L. V. Prasad Eye Institute, Hyderabad, IND; 3 Department of Vitreoretinal Diseases/Tele-Ophthalmology, L. V. Prasad Eye Institute, Hyderabad, IND

**Keywords:** emr, diabetic retinopathy, big data, india, covid-19 pandemic

## Abstract

Objective: This study aimed to describe the demographics and clinical profile of patients with diabetic retinopathy (DR) presenting during the novel coronavirus disease 2019 (COVID-19) lockdown and unlock phases in India.

Methods: This hospital-based cross-sectional study included patients presenting from March 25, 2019, to March 31, 2021. All patients who presented with DR were included as cases. The data were collected using an electronic medical record system.

Results: In total, 88,012 patients diagnosed with retinal diseases were presented to the network and included for analysis. There were 21,271 (24%) DR patients during the study period and the majority were men (71%) from the urban area (45%). An increasing number of patients with proliferative DR (56%), sight-threatening DR (79%), need for vitreoretinal procedures (31%), and intravitreal injections (19%) were seen during the lockdown (phase one-four). There was a significant increase in the number of patients with blindness in pre-lockdown (20%), lockdown (32%), and post-lockdown (26%). Patterns of sight-threatening DR and blindness were similar in both fresh and follow-up patients.

Conclusion: The presentation of DR patients in hospital is evolving because of the COVID-19 pandemic. The footfalls of patients during the unlock (phase 1-10) regained to two-thirds of the pre-COVID-19 level. There was an increase in patients with sight-threatening DR and the need for vitreoretinal surgery and intravitreal injections during the lockdown (phase 1-4).

## Introduction

The ongoing novel coronavirus disease 2019 (COVID-19) pandemic has changed the world, as we know it affecting more than 141 million individuals [1]. The past year has seen unprecedented nationwide lockdowns of populations that altered the curve of the COVID-19 cases. The Government of India enforced policies to prevent the spread of the COVID-19 virus in a population of 1.3 billion people [2]. Studies have shown that access to patient care showed a sharp decline during the lockdown period in India and that a vitreoretinal procedure was required in a third of the patients who underwent a surgical intervention [3,4]. The unlock 1.0 guidelines in India that were released from June 2020 ensured unrestricted movement of persons and goods [5]. Rathi et al. shared the experience of unlock 1.0 on eyecare services which showed the highest reduction of patient footfalls in urban centers and there was no significant change in the uptake of services by gender [6]. Hanumanthadu et al. utilizing a retina diagnostic hub reported that the most common diagnosis made was diabetic retinopathy [7]. The consensus guidelines that were published for vitreoretinal diseases outlined triaging patients into urgent, semi-urgent, and delayed appointments based on the complaints and nature of the disease, provided guidelines on retinal examination techniques, retinal imaging, and prioritizing vitreoretinal surgery into emergency, semi-emergency, and elective procedures [8]. While the world is adapting to the new normal in the delivery of healthcare services, it is important to understand the trends in access to eyecare in situations such as this ongoing pandemic to identify the vulnerable groups in the population for corrective action. The authors describe a comparative report of the impact on the presentation of patients with diabetic retinopathy and other retina diseases to a large multitier ophthalmology network in India during the ongoing COVID-19 pandemic.

## Materials and methods

Study design, period, location, and approval

This cross-sectional observational hospital-based study included all patients diagnosed with retinal disorders presenting between March 25, 2019, and March 31, 2021, to a multitier ophthalmology network located in India [9]. A standard consent form for electronic data privacy was signed by the patient or the parents or guardians of the patient at the time of registration. None of the identifiable parameters of the patient information were used for analysis of the data. The study adhered to the declaration of Helsinki and was approved by the Institutional Ethics Committee (LEC BHR-R-05-21-659). The clinical data of each patient who underwent a comprehensive ophthalmic examination was entered into a browser-based electronic medical records system (eyeSmart EMR {L V Prasad Eye Institute, Hyderabad, Telangana}) using a standardized template by trained ophthalmic personnel and supervised by an ophthalmologist [10].

Data retrieval and processing 

A total of 88,012 patients of all ages diagnosed with retinal disorders presented to the network during the study period and were included in this study. The data of these patients were retrieved from the electronic medical record database and segregated in a single excel sheet (Microsoft Excel 2019 {Microsoft Corporation, Redmond, WA}). Data on patient demographics, clinical presentation, ocular diagnosis, and treatment modalities were used for analysis. The excel sheet with the required data was then used for analysis using the appropriate statistical software (Stata Statistical Software {College Station, TX: StataCorp LLC.}). Standardized definitions were used for occupation, socio-economic status, and geographic distribution [11]. The visual acuity was classified as mild or no visual impairment (20/20 to 20/70), moderate visual impairment (>20/70 to 20/200), severe visual impairment (>20/200 to 20/400), blindness category 3 (>20/400 to 20/1200), blindness category 4 (>20/1200 to perception of light), and blindness category 5 (no perception of light) according to the WHO guidelines [12]. The study duration was divided into three categories, pre-COVID-19 between March 25, 2019, and March 24, 2020, lockdown (phase 1-4) between March 25, 2021, and May 31, 2021, and unlock (phase 1-10) between June 1, 2020, and March 31, 2021 [13]. The geographic distance was classified in relation to the eye care center at presentation. The patients presenting from the same location of the eye center were classified as “intracity,” those from the same state of the eye center were classified as “intrastate,” and the rest of the patients were classified as “interstate.” A subset analysis was performed on 21,271 patients who were diagnosed with diabetic retinopathy. The clinical features of severe non-proliferative diabetic retinopathy (NPDR)/proliferative diabetic retinopathy (PDR)/diabetic macular edema (DME) were classified as sight-threatening diabetic retinopathy (STDR) [14]. The demographic distribution and clinical presentation of the patients in these three categories were used for comparative analysis.

Statistical analysis

Descriptive statistics using mean±standard deviation and median with interquartile range (IQR) were used to elucidate the demographic and clinical data using Microsoft Excel 2019.

## Results

Overall, 88,012 patients diagnosed with retinal diseases presented during the study period. The most common retinal disease was diabetic retinopathy in 21,271 (24%) patients, optic nerve-related pathology in 7135 (8%), retinal detachment in 6812 (8%) patients, and vein occlusions in 6100 (7%) patients.

Diabetic retinopathy

Overall, there were 22,399 patients with diabetes mellitus who presented during the study period, of which 13,172 (59%) patients presented during pre-COVID phase, 458 (2%) patients during the lockdown, and 8769 (39%) presented during the unlock phase. Overall, 21,271 patients diagnosed with diabetic retinopathy presented during the study period. The overall mean number of patients seen per day was 28.8 (21,271/738). The patients seen during the lockdown phase were significantly lower with a mean of 6.7 (456/68) compared to the pre-COVID-19 phase with a mean of 33.5 (12,285/366) and increased to a mean of 28.1 (8530/303) during the unlock phase. The mean age of the patients was 56.59±9.44 years while the median was 57 (IQR: 50-63) years. There were four (0.02%) patients who were children (≤16 years) and 21,267 (99.9%) were adults. The most common age group at presentation was between 51 and 60 years with 8663 (40.7%) patients. Majority were male 15,045 (70%) and 6226 (30%) female patients.

There were 9616 (45%) patients from the urban districts, 8799 (42%) from rural districts, and 2856 (13%) from metropolitan regions. With regards to geographic distance, 6132 (29%) patients presented from the intracity region, 10,950 (51%) patients from the state, and 4189 (20%) from outside the state. There were 18,403 (87%) patients from the paying category and 2868 (13%) from the nonpaying category. The patients presented more commonly from the states of Andhra Pradesh with 7177 (34%) patients, Telangana with 7009 (33%) patients, followed by Odisha with 4400 (21%) patients. The most common form of diabetic retinopathy was sight-threatening diabetic retinopathy (STDR) seen in 13,875 (65%) patients, and non-sight threatening DR (NSTDR) was seen in 7396 (35%) of patients. A majority of STDR patients were fresh patients (10,914 {79%}) and 2961 (21%) were follow-up patients.

Effect of Pandemic on DR

Among the patients with diabetic retinopathy, there was an increasing trend seen during the lockdown phase with males (75%), paying category (90%), and in patients from the intracity region (31%). There was a decreasing trend seen in females (25%), nonpaying category (11%), and patients presenting from outside the state (15%).

**Figure 1:**
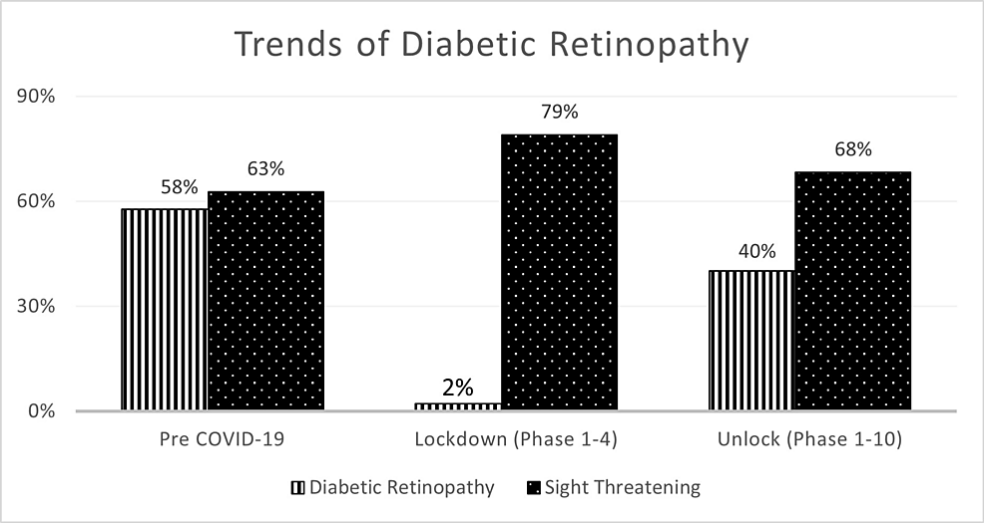
Comparison of trends of patients with DR and STDR during the pre-COVID-19, lockdown (phase 1-4), and unlock (phase 1-10) DR: diabetic retinopathy; STDR: sight-threatening diabetic retinopathy; COVID 19: coronavirus disease 2019

Among the patients with diabetic retinopathy, there was an increase in patients seen with proliferative diabetic retinopathy (56%; p≤0.00001) and sight-threatening diabetic retinopathy (79%; p=0.001) and a decrease in patients with non-proliferative diabetic retinopathy (25%; p≤0.00001) during the lockdown phase (Figure 1). The increase in the STDR patients was seen across the pre-COVID, lockdown, and unlock phases (Table 1).

**Table 1:** Comparison of baseline characteristics during the pre-COVID period, lockdown (phase 1-4), and unlocking (phase 1-10) for diabetic retinopathy patients. *As compared to pre-COVID-19 DR: diabetic retinopathy; NPDR: non-proliferative diabetic retinopathy; PDR: proliferative diabetic retinopathy; DME: diabetic macular edema; STDR: sight-threatening diabetic retinopathy; NSDR: non-sight threatening diabetic retinopathy; COVID 19: coronavirus disease 2019

Variable		n	%	Pre-COVID-19	%	Lockdown (phase 1-4)	%	Unlock (phase 1-10)	%	p-Value*
Total DR		21,271	100	12,285	58	456	2	8530	40	
Gender	Male	15,045	71	8570	70	342	75	6133	72	0.32
	Female	6226	29	3715	30	114	25	2397	28	0.07
Age (years)	0-30	150	0.7	76	0.6	5	1	69	0.8	0.21
	31-40	760	3.6	411	3	26	6	323	4	0.009
	41-50	4464	21	2476	20	117	26	1871	22	0.022
	51-60	8663	41	4949	40	187	41	3527	41	0.84
	61-70	5863	28	3522	29	102	22	2239	26	0.025
	71-100	1371	6	851	7	19	4	501	6	0.030
Socio-economic status	Paying	18,403	87	10,399	85	408	90	7596	89	0.42
	Nonpaying	2868	13	1886	15	48	10	934	11	0.013
Geographic status	Urban	9616	46	5699	47	197	43	3720	44	0.41
	Rural	8799	41	4939	40	182	40	3678	43	0.93
	Metropolitan	2856	13	1647	13	77	17	1132	13	0.06
Distance to eye care center	Intracity	6132	29	3282	27	189	41	2661	31	<0.00001
	Intrastate	10,950	51	6087	50	235	52	4628	54	0.63
	Interstate	4189	20	2916	24	32	7	1241	15	<0.00001
Type of DR	NPDR	8675	41	5343	44	116	25	3216	38	<0.00001
	PDR	8170	38	4430	36	255	56	3485	41	<0.00001
	DME	4426	21	2512	20	85	19	1829	21	0.44
	STDR	13,875	65	7697	63	360	79	5818	68	0.001
	NSTDR	7396	35	4588	37	96	21	2712	32	<0.00001

Treatment Interventions

Among treatment interventions, there were 5091 (24%) patients who underwent vitreoretinal surgery and 3016 (14%) patients who were given intravitreal injections. A detailed comparison of all three phases is described in Table 2.

**Table 2:** Comparison of visual impairment and interventions during the pre-COVID period, lockdown (phase 1-4), and unlocking (phase 1-10) for diabetic retinopathy patients *As compared to pre-COVID-19 COVID 19: coronavirus disease 2019

Variable		n	%	Pre-COVID-19	%	Lockdown (phase 1-4)	%	Unlock (phase 1-10)	%	p-Value*
Visual impairment	Mild/No	9083	42	5618	46	177	39	3288	39	<0.00001
	Moderate	4335	20	2487	20	75	16	1763	21	0.85
	Severe	1905	9	1063	9	35	8	807	9	0.09
	Blind	4907	23	2547	21	144	32	2216	26	<0.00001
	Unspecified	1041	5	560	5	25	5	456	5	0.01
Interventions	Vitreoretinal surgery	5091	24	2701	22	167	37	2223	26	<0.00001
	Intravitreal injections	3016	14	1595	13	86	19	1335	16	0.001

Visual Impairment and Blindness due to DR

The average logMAR decreased from 1.09±1.07 in the pre-COVID phase to 1.48±1.1 during the lockdown phase and improved to 1.24±1.1 in the unlock phase. Table 2 shows visual impairment and blindness trends among people with DR. Approximately 20% of blindness was observed before lockdown and this percentage increased to 32% during the lockdown phase and the upward trend continued with 26% blindness after the lockdown period (p<0.0001) (Table 2). These rising trends in blindness were distributed equally between fresh/follow-up patients (Table 3).

**Table 3:** Comparison of STDR, NSTDR, visual impairment, and blindness during the pre-COVID period, lockdown (phase 1-4), and unlocking (phase 1-10) for fresh and follow-up diabetic retinopathy patients NPDR: non-proliferative diabetic retinopathy; STDR: sight-threatening diabetic retinopathy

Patient details		n	%	Pre-COVID-19	%	Lockdown (phase 1-4)	%	Unlock (phase 1-10)	%	p-Value
Fresh patients	STDR	10,914	79	6020	78%	256	71	4638	80%	0.25
	NSTDR	5708	77	3554	77%	73	76	2081	77%	0.90
Visual impairment	Mild/No	6513	39	4062	42	119	36	2332	35	0.14
	Moderate	3731	22	2135	22	63	19	1533	23	0.27
	Severe	1631	10	902	9	30	9	699	10	0.86
	Blindness	3890	23	2004	21	99	30	1787	27	0.001
Follow-up patients	STDR	2961	21	1677	22	104	29	1180	20	0.013
	NSTDR	1688	23	1034	23	23	24	631	23	0.79
Visual impairment	Mild/No	2570	55	1556	57	58	46	956	53	0.15
	Moderate	604	13	362	13	12	9	230	13	0.25
	Severe	274	6	161	6	5	4	108	6	0.37
	Blindness	1017	22	543	20	45	35	429	24	0.001

## Discussion

This study sought to describe the demographics and clinical profile of patients with diabetic retinopathy presenting during the pre-COVID-19, lockdown phase, and unlock phase in India. The findings of this study suggest that the mean footfalls of patients with diabetic retinopathy showed a sharp decline during the lockdown phase and regained to two-thirds of the pre-COVID-19 level during the unlock phase.

There was an increasing trend seen in males, higher socio-economic status and presentation from the local region. There was a decreasing trend seen in females, lower socio-economic status and presentation from outside the state. These findings suggest the need to find ways to reach patients of lower socio-economic status and women. As was the case with our study, Kavitha Singh et al. reported that people with chronic diseases, especially among the poor, rural and marginalized, have experienced challenges accessing health care [15]. We are also planning other care portals such as teleophthalmology to provide guidance and care in coordination with local ophthalmologists to patients outside the state in the future.

In an earlier study published by the same authors of their experience of patient footfalls during the lockdown, medical retina accounted for 20% and vitreoretinal procedures accounted for 14% of the emergency cases (65%) seen in the outpatient department. About a third of the patients (33%) underwent a vitreoretinal surgical intervention and the most common procedure was a retinal detachment surgery (48%) [3]. In this study, diabetic retinopathy (24%) was the most common etiology among all retinal diseases presented in hospital. The majority were patients with STDR (79%) among fresh and follow-up patients.

A simulated prediction model on impact of the COVID-19 pandemic on diabetes complications in India predicted increase in risks of 2.8% for non-proliferative diabetic retinopathy, 2.9% for proliferative diabetic retinopathy, 1.5% for retinal photocoagulation. The model showed that the duration of lockdown is directly proportional to the worsening of glycemic control and diabetes-related complications [16]. In the present study, compared to pre-lockdown phase (63%), a significant increase in number of fresh and follow-up patients presented with STDR during lockdown (79%) and post-lockdown (68%). Vitreoretinal surgical procedures showed an upward trend with respect to pre-lockdown (22%), lockdown (37%), and post-lockdown (26%). Intravitreal injections were also higher with respect to pre-lockdown (13%), lockdown (19%), and post-lockdown (16%). These increasing trends in STDR and treatment trends clearly indicate the adverse impact of the pandemic on patients with DR.

Unlike our study, studies in the United States (down 9.9%) and France reported declining trends in intravitreal injections (down 11.5%) after the lockdown period [17,18]. In a recent systematic review on COVID-19 pandemic on DR monitoring and treatment revealed that intravitreal injections for DR have decreased significantly globally during the lockdown period of pandemic, ranging from approximately 30% to nearly 100% reduction, compared to corresponding time points in 2019 [19].

We observed a significant increase in patients presenting with visual impairment and blindness with respect to pre-lockdown (20%), lockdown (32%), and post-lockdown (26%). Similar negative impact of a pandemic on DR was observed in a study conducted from Greece. They reported an increased worsening of DR to active PDR post-lockdown. Postponement of care during pandemic was found to be the significant factor for worse visual outcomes [20]. In DME cases, there were unanticipated treatment delays due to lockdown that resulted in worsening and visual impairment [21]. Similar negative visual outcomes in patients with DME following pandemic have been reported from China and the United States [22,23]. Tele-screening, home monitoring systems such as the Alleye app (Oculocare Medical AG, Zurich, Switzerland), portable OCT (Duke University, Durham, NC) for DME detection are some of the innovative methodologies reported to reduce the adverse impact of a pandemic on DR management outcomes [24-26].

Patients with diabetes who are often comorbid are at high risk of contracting COVID-19. Hospital quality control teams have developed appropriate protocols to handle an increased number of interventions during the study to minimize the risk of infection [27]. Same-day injections, surgeries, and post-operative examinations were part of the effort to minimize outpatient visits [28]. An integrated web-based teleconsultation portal has been established to handle post-operative follow-up consultations via teleconsultation [29]. Patients were encouraged to consult with local physicians for post-operative eye examinations and an assessment of intraocular pressure. They were able to upload those reports during teleconsultation. By reducing one or more follow-up visits, it was possible to reduce decongestion in patient waiting rooms and implement social distancing protocols and reduce wait times.

The study limits include the retrospective nature of the study. There is a lack of data on systemic/biochemical endpoints for direct correlation of DR severity with systemic control of DM. The results of the study suggest the need for eye care facilities to prepare for the presentation of patients with sight-threatening DR with significant blindness which will require more treatment procedures during a pandemic.

## Conclusions

In conclusion, the authors present their experience on the demographic and clinical presentation of patients with diabetic retinopathy presenting to a multi-tier ophthalmology network in India during the COVID-19 pandemic. The vulnerable patient groups of females, lower socio-economic status, and patients traveling from outside the state need focused attention in times of crisis such as this. The footfalls of patients during the unlock phase regained to two-thirds of the pre-COVID-19 level. There was an increase in patients with sight-threatening diabetic retinopathy and the need for vitreoretinal surgery and intravitreal injections during the lockdown phase.
